# Correlation Between Coronary Artery Disease Severity Detected by CT Coronary Angiography and Grade of Left Ventricular Diastolic Dysfunction Detected by Echocardiography

**DOI:** 10.3390/jcm14207218

**Published:** 2025-10-13

**Authors:** Ahmed El-Barbary, Mohamed Atef Elsayed, Yousef Ahmed Yousef Selim, Sameh Mohamed Helmy Elkaffas, Hussein Sabit, Borros Arneth, Zulfugar T. Taghiyev, Mahmoud Ahmed Tantawy

**Affiliations:** 1 Department of Cardiology, Faculty of Medicine, Misr University for Science and Technology, Giza P.O. Box 77, Egypt; atefalabd66@gmail.com (M.A.E.);; 2 Department of Radiology, Faculty of Medicine, Misr University for Science and Technology, Giza P.O. Box 77, Egypt; 3 Department of Medical Biotechnology, College of Biotechnology, Misr University for Science and Technology, Giza P.O. Box 77, Egypt; 4 Institute of Laboratory Medicine and Pathobiochemistry, Molecular Diagnostics, Hospital of the Universities of Giessen and Marburg (UKGM), Justus Liebig University Giessen, 35392 Giessen, Germany; borros.arneth@klinchemie.med.uni-giessen.de; 5 Department of Cardiovascular Surgery, Hospital of the Universities of Giessen and Marburg (UKGM), Justus Liebig University Giessen, 35392 Giessen, Germany

**Keywords:** coronary artery disease, diastolic dysfunction, CT coronary angiography, echocardiography, segment involvement score (SIS), coronary artery calcium score (CACS)

## Abstract

**Background:** Coronary artery disease (CAD) and left-ventricular (LV) diastolic dysfunction are leading drivers of morbidity and mortality. Clarifying how anatomical CAD burden relates to diastolic impairment may refine diagnosis and risk stratification. **Methods:** We conducted a cross-sectional analytical study of 200 adults with intermediate pretest probability of CAD who underwent both coronary CT angiography (CCTA) and transthoracic echocardiography (TTE) within ≤1 year. Coronary burden was quantified by Segment Involvement Score (SIS). Diastolic function was graded by contemporary echocardiographic guidelines. Patients were classified as obstructive (≥50% LM or ≥70% in other major epicardial arteries) or non-obstructive CAD. **Results:** Obstructive CAD was present in 73/200 (36.5%). Diastolic dysfunction occurred in 161/200 (80.5%) and was markedly more prevalent/severe in obstructive vs. non-obstructive CAD (*p* < 0.001). SIS rose stepwise with higher diastolic dysfunction grades; SIS correlated strongly with diastolic grade (r = 0.809, *p* < 0.001). Compared with non-obstructive CAD, obstructive CAD showed worse diastolic indices (higher E/e′, larger LAVI, shorter DT and IVRT; all *p* < 0.001) and a shift toward Grades II–III. Obstructive CAD was also associated with higher total cholesterol, triglycerides, LDL, HbA1c, lower HDL (all *p* ≤ 0.002), and a greater prevalence of hypertension and diabetes. **Discussion:** Increasing coronary atherosclerotic burden—captured by SIS, parallels progressive impairment of LV relaxation and elevated filling pressures, supporting a pathophysiologic link between epicardial disease extent and diastolic dysfunction. **Conclusions**: In symptomatic intermediate-risk patients, greater CAD extent on CCTA is strongly associated with higher grades of LV diastolic dysfunction on echocardiography. Integrating anatomic (SIS) and functional (TTE) metrics may enhance risk assessment and guide management in CAD.

## 1. Introduction

Cardiovascular disease (CVD), particularly (CAD), remains a leading cause of morbidity and mortality worldwide, posing a substantial burden on both men and women [1]. Coronary artery disease is a multifactorial condition and is one of the leading causes of death in the world [2]. In 2022 alone, coronary heart disease accounted for over 370,000 deaths in the United States, highlighting its continued significance as a public health crisis [3,4]. Although traditionally perceived as a predominantly male disease, CAD is equally critical in women, who often present with different symptom profiles and experience higher rates of misdiagnosis and adverse outcomes [5]. This gender disparity is partially due to gaps in awareness, risk stratification, and the interpretation of clinical presentation across sexes.

Typical symptoms of CAD include central oppressive chest pain, jaw and left arm pain, diaphoresis, shortness of breath, nausea, and vomiting [6]. However, a significant proportion of patients—particularly women and those with diabetes—present with atypical symptoms such as fatigue, epigastric pain, dull or burning chest discomfort, palpitations, lightheadedness, neck or back pain, indigestion, right arm or shoulder discomfort, dizziness, or even syncope [7,8]. These non-classical manifestations often result in under-recognition of CAD in clinical practice, which can delay diagnosis and appropriate intervention. The failure to recognize these presentations can lead to missed diagnoses, under-treatment, and ultimately worse patient outcomes, emphasizing the need for a more inclusive and nuanced understanding of symptomatology in CAD.

A study suggests that labeling symptoms as ‘typical’ or ‘atypical’ should no longer be done, as the presentation of CAD varies significantly with age, sex, race, and other preexisting risk factors [8]. This variability underscores the limitations of rigid clinical heuristics and supports a shift towards personalized assessment strategies incorporating demographic and biological heterogeneity. Increasing evidence suggests that symptom interpretation must be contextualized within patient-specific risk profiles to ensure accurate diagnosis and management [9].

In terms of diagnosis, (CCTA) is increasingly recommended as a first-line investigation for establishing the presence of coronary artery disease [10]. CCTA has gained prominence due to its noninvasive nature, high negative predictive value, and ability to visualize both obstructive and non-obstructive plaque burden directly. Recent expert consensus and guidelines, such as those issued by the Society of Cardiovascular Computed Tomography, have recognized CCTA as an essential modality for initial risk stratification in patients presenting with chest pain [11]. Moreover, its integration with advanced techniques such as CT-derived fractional flow reserve (FFR-CT) enhances its capacity to assess the functional significance of coronary lesions, improving diagnostic precision.

The assessment of left ventricular (LV) diastolic function is also essential to the comprehensive evaluation of cardiac function through echocardiography. The American Society of Echocardiography/European Association of Cardiovascular Imaging guidelines recommend the inclusion of multiple measurements, as well as considering underlying myocardial pathology, such as impaired LV long-axis systolic function, increased LV end-diastolic pressure (evidenced by pulmonary venous flow), and pathological hypertrophy [12]. These parameters enable clinicians to grade diastolic dysfunction with higher specificity and are especially valuable in patients with preserved ejection fraction but ongoing symptoms suggestive of ischemia.

Notably, the interrelationship between CAD and diastolic dysfunction has emerged as a topic of growing research interest. Several studies have suggested that diastolic dysfunction may not only co-occur with CAD but may also be exacerbated by ischemic burden and microvascular dysfunction [13]. Subclinical ischemia can impair myocardial relaxation and compliance, thereby contributing to the development of heart failure with preserved ejection fraction (HFpEF)—a condition associated with high morbidity and limited treatment options [12]. Furthermore, the impact of coronary atherosclerosis on myocardial tissue composition, particularly in terms of fibrotic remodeling and reduced compliance, has been implicated in the pathogenesis of diastolic impairment [14].

The correlation between the severity of CAD detected by CCTA and the grade of LV diastolic dysfunction detected by echocardiography remains an area of clinical importance. Establishing this relationship could enable clinicians to better risk-stratify patients and identify those at greater risk of progression to HFpEF. It could also foster more integrated use of multimodal imaging in routine cardiac evaluations, thus aligning diagnostic workflows with the complexities of modern cardiovascular pathophysiology. Recognizing and quantifying the interplay between anatomical disease burden and functional myocardial impairment may ultimately lead to more tailored and effective therapeutic strategies.

The study aims to determine the correlation between the severity of coronary artery disease detected by CT coronary angiography and the grade of LV diastolic dysfunction detected by echocardiography.

## 2. Patients and Methods

### 2.1. Study Design and Population

This cross-sectional analytical study was carried out at Misr University for Science and Technology Teaching Hospital and Alfa Scan Laboratories from November 2022 to November 2024. This study included 200 patients who underwent both coronary computed tomography angiography (CTCA) and transthoracic echocardiography (TTE), with a maximum of one year between the two tests. The mean (± SD) interval between CTCA and TTE was 6.2 ± 3.0 months, with a median of 6 months (interquartile range [IQR] 3–9 months).

#### Recruitment Flow

A total of 250 patients were screened for eligibility. Of these, 50 patients were excluded due to not meeting the inclusion criteria (e.g., LVEF < 45%, moderate or severe valvular disease, primary myocardial pathologies, significant pericardial diseases, congenital heart disease, primary pulmonary hypertension, atrial or ventricular arrhythmias, or contraindications to CTCA). Thus, 200 patients were included in the study.

### 2.2. Inclusion and Exclusion Criteria

This study included adult patients aged 20 years or older, encompassing both genders, who provided informed written consent and presented with an intermediate pretest probability of coronary artery disease, as determined by the Pretest Probability Formula accounting for age, sex, chest pain characteristics, and history of diabetes, hypertension, dyslipidemia, and smoking. Patients were excluded if they declined participation, had a left ventricular ejection fraction (LVEF) below 45%, suffered from moderate or severe valvular disease, or were diagnosed with primary myocardial pathologies such as hypertrophic or restrictive cardiomyopathy. Further exclusions applied to individuals with significant pericardial diseases like constriction, congenital heart disease, primary pulmonary hypertension, those experiencing atrial or ventricular arrhythmias at the time of imaging, or those with contraindications to coronary CT angiography testing.

### 2.3. Methods

After obtaining informed written consent from each participant, a comprehensive data collection process was initiated. This involved detailed history taking, encompassing personal details (age, sex, weight), cardiovascular risk factors (smoking status, hypertension, diabetes mellitus, dyslipidemia), relevant surgical history including previous coronary artery bypass grafting (CABG) or percutaneous coronary intervention (PCI) and valve surgeries, and family history of cardiovascular disease. A thorough clinical examination was conducted, including assessment of vital signs (pulse, blood pressure, respiratory rate, temperature), measurement of weight and height for Body Mass Index (BMI) calculation, and a focused cardiovascular examination to detect any abnormal heart sounds, murmurs, or pericardial rubs. Laboratory investigations included a complete blood count (CBC) assessing hemoglobin, white blood cell count, and platelet count; glycated hemoglobin (HbA1c); liver enzymes (ALT, AST); a complete lipid profile (total cholesterol, triglycerides, LDL, HDL); and renal function tests (serum creatinine, urea). Transthoracic echocardiography was performed according to the American Society of Echocardiography (ASE) and European Association of Cardiovascular Imaging (EACVI) guidelines, utilizing standard M-mode, 2D, and Doppler techniques. Left ventricular diastolic function was evaluated explicitly by measuring the ejection fraction (EF), mitral inflow early (E) to late (A) filling velocity ratio (E/A), peak tricuspid regurgitation (TR) velocity, septal and lateral mitral annular early diastolic velocities (e′) via tissue Doppler imaging, the E/e′ ratio, mitral E-wave deceleration time (DT), isovolumic relaxation time (IVRT), and the left atrial (LA) volume index, which was determined using Simpson’s rule from the 4-chamber view and indexed to body surface area. Contrast-enhanced CT coronary angiography was used to identify patients with obstructive coronary artery disease. For the purposes of this study we defined obstructive CAD as ≥50% stenosis in the left main coronary artery or ≥70% stenosis in any other major epicardial coronary artery. This unified definition was applied consistently throughout the analysis to dichotomize patients into obstructive and non-obstructive cohorts.

### 2.4. Left Ventricular Diastolic Function

Left ventricular diastolic function was graded based on specific echocardiographic parameters derived from the transthoracic examination. Normal diastolic function was defined by a septal e’ velocity > 8 cm/s, lateral e′ velocity > 10 cm/s, left atrial (LA) volume index < 34 mL/m^2^, mitral inflow E/A ratio < 0.8, mitral E-wave deceleration time (DT) > 200 ms, average E/e′ ratio < 8, pulmonary vein atrial reversal wave duration minus mitral A-wave duration (Ar-A duration) < 0 ms, and a change in E/A ratio with Valsalva maneuver (ΔE/A) of < 0.5. Grade 1 diastolic dysfunction (Impaired Relaxation) was characterized by septal e′ < 8 cm/s, lateral e′ < 10 cm/s, LA volume index > 34 mL/m^2^, E/A ratio < 0.8, DT > 200 ms, average E/e′ ratio between 9 and 12, Ar-A duration > 30 ms, and Valsalva ΔE/A < 0.5. Grade 2 diastolic dysfunction (Pseudonormal Filling) was identified by septal e′ < 8 cm/s, lateral e′ < 10 cm/s, LA volume index > 34 mL/m^2^, E/A ratio between 0.8 and 1.5, DT between 160 and 200 ms, average E/e′ ratio > 13, Ar-A duration > 30 ms, and Valsalva ΔE/A > 0.5. Finally, Grade 3 diastolic dysfunction (Restrictive Filling) was determined by septal e′ < 8 cm/s, lateral e′ < 10 cm/s, LA volume index > 34 mL/m^2^, E/A ratio ≥ 2, DT < 150 ms, average E/e’ ratio > 15, Ar-A duration > 40 ms, and Valsalva ΔE/A > 0.5.

### 2.5. Coronary Artery Calcium Score (CACS)

Plain coronary multi-slice CT to assess coronary artery calcium score (CACS), providing a noninvasive quantitative evaluation of coronary atherosclerotic burden, which enables stratification of cardiovascular risk, prognostication, and guides preventive therapeutic interventions in asymptomatic and symptomatic individuals. In the absence of contraindications to coronary multi-slice CT, premedication with an oral beta blocker were given in case of heart rate was greater than or equal to 70 beats/min. Additional oral ivabradine (7.5 mg twice daily for 3 days before the examination) were added if tolerated. The Agatston scoring method was used to determine the calcium score in the coronary arteries. Total Agatston score was measured in patients with a positive CACS that was less than 400. Coronary plaques types were classified visually by using contrast-enhanced CT coronary angiography to identify luminal area stenosis as normal, non-obstructive (<50% in the left main stem or <70% in other segments) or obstructive (≥50% in the left main stem or ≥70% in other segments), and classified quantitively according to Mean Hounsfield units (HU) densities which ranged from 14 to 75 HU for lipid-rich soft plaques 67 to 149 HU for fibrous plaques and 135 to 1089 HU for calcified plaques the extent of coronary atherosclerosis was quantified using the Segment Involvement Score (SIS) derived from CT Coronary Angiography. The SIS provides a semi-quantitative measure of overall plaque burden by summing the total number of coronary artery segments (maximum 17) exhibiting any detectable atherosclerotic plaque, irrespective of individual plaque characteristics or the degree of stenosis within the affected segment. This score aligns with standardized quantitative CTA reporting frameworks like CAD-RADS, facilitating consistent assessment of disease extent.

Building upon the Segment Involvement Score (SIS) calculation, which quantifies the number of coronary segments with detectable plaque based on a standard 17-segment model, the total score was utilized to categorize the overall coronary plaque burden according to the CAD-RADS 2.0 system’s P-modifier categories. Specifically, an SIS (representing the sum of involved segments) of ≤2 corresponded to mild plaque burden (P1), SIS 3–4 indicated moderate burden (P2), SIS 5–7 denoted severe burden (P3), and an SIS ≥ 8 signified extensive plaque burden (P4). This categorization is clinically relevant, as a higher SIS is recognized as an independent predictor associated with an increased probability of adverse cardiovascular events. Furthermore, the SIS provides a framework for risk stratification: an SIS of 0 suggests no plaque involvement, scores from 1 to 5 generally indicate mild involvement associated with lower risk, while scores greater than 5 imply moderate to severe plaque burden, correlating with an increased risk for adverse cardiac outcomes.

### 2.6. Ethical Considerations

This study was conducted following approval from the Research Ethics Committee at the Faculty of Medicine, Misr University for Science and Technology (MUST). Prior to enrollment, informed written consent was obtained from all participating patients or their first-degree relatives; the consent process included providing detailed information regarding the study’s aim, design, methodology, location, duration, and assurance of data confidentiality. Furthermore, official permission to conduct the research was secured from both the Dean of the Faculty of Medicine at MUST and the administration of Alfa Medical Group.

### 2.7. Sample Size Calculation

This study was based on a study by [14]. Epi Info STATCALC was used to calculate the sample size by considering the following assumptions: −95% two-sided confidence level, with a power of 80% and an error of 5% odds ratio calculated = 1.115. The final minimum sample size taken from the Epi-Info output was 100 [14].

### 2.8. Statistical Analysis

Collected data were organized, coded, tabulated, and statistically analyzed using IBM SPSS Statistics software (version 27, IBM Corp., Armonk, NY, USA). The normality of data distribution was evaluated using methods such as the Shapiro–Wilk test and histogram inspection. Quantitative parametric data were presented as mean and standard deviation (SD); comparisons between two groups utilized Student's *t*-test, while comparisons among multiple groups employed ANOVA (F-test) with post hoc analysis (e.g., Tukey test). Quantitative non-parametric data were presented as median and interquartile range (IQR) and analyzed using the Kruskal–Wallis test for multi-group comparisons, followed by the Mann–Whitney U test for pairwise comparisons between groups. Categorical variables were presented as frequencies and percentages (%), and differences between groups were assessed using the Chi-square test. Pearson’s correlation coefficient (r) was calculated to evaluate correlations between variables. A two-tailed *p* value < 0.05 was considered statistically significant for all analyses.

Data on current cardiovascular medications (including statins, beta-blockers, ACE inhibitors and antiplatelet therapy) were collected during history taking. These variables were evaluated as potential covariates in preliminary models; however, none significantly modified the associations reported. To avoid over-fitting, medication variables were therefore not included in the final multivariate analyses.

## 3. Results

This cross-sectional analytical investigation encompassed 200 patients who underwent both (CCTA) and (TTE), ensuring an interval of no more than one year between the two diagnostic assessments; the detailed baseline demographic and clinical characteristics of the study cohort are comprehensively summarized in Table 1.

Regarding the baseline characteristics, the age of the studied patients ranged from 40 to 73 years with a mean (±SD) of 54.27 ± 8.98 years; 104 (52%) patients were males, and 96 (48%) patients were females. The weight ranged from 55 to 95 kg with a mean (±SD) of 74.83 ± 12.45 kg, the height ranged from 1.58 to 1.75 m with a mean (±SD) of 1.68 ± 0.05 m, and the BMI ranged from 18.29 to 36.65 kg/m^2^ with a mean (±SD) of 26.73 ± 4.73 kg/m^2^.

### 3.1. Risk Factors of the Studied Patients

The study included a total of 200 participants. Among these individuals, the prevalence of key risk factors was evaluated. Smoking was reported by 52 participants (26%). A relevant family history was noted in 43 participants (21.5%). Hypertension was observed in 80 individuals (40%). Furthermore, 57 participants had Diabetes Mellitus (DM) (28.5%), and 66 participants had dyslipidaemia (33%).

### 3.2. Baseline Laboratory Findings

Baseline laboratory investigations were performed. For example, the mean (±SD) hemoglobin concentration was 12.33 ± 1.14 g/dL, mean HbA1c was 5.23 ± 1.51%, and mean total cholesterol was 228.78 ± 58.1 mg/dL. Full details of all laboratory findings are presented in Table 2.

### 3.3. Baseline Echocardiographic Findings

Baseline echocardiographic findings were also assessed. For instance, the mean (± SD) ejection fraction (EF) was 59.28 ± 5.03%, and the mean left atrial volume index (LAVI) was 31.85 ± 3.74 mL/m^2^. Regarding diastolic function, 39 patients (19.5%) had normal function, while 82 (41%), 58 (29%), and 21 (10.5%) had grade I, II, and III diastolic dysfunction, respectively. Full details are available in Table 3.

Regarding the echocardiographic findings, EF ranged from 49 to 69% with a mean (±SD) of 59.28 ± 5.03%. LAVI ranged from 26 to 43 mL/m^2^ with a mean (±SD) of 31.85 ± 3.74 mL/m^2^. Septal e′ ranged from 0.5 to 1.1 m/s with a mean (±SD) of 0.83 ± 0.17 m/s. Lateral e′ ranged from 0.03 to 0.25 m/s with a mean (±SD) of 59.28 ± 5.03 m/s. E/e′ ranged from 49 to 69 with a mean (±SD) of 0.12 ± 0.05. E/A ranged from 0.71 to 2.55 with a mean (±SD) of 1.39 ± 0.44. TR velocity ranged from 0.8 to 3.1 m/s with a mean (±SD) of 1.85 ± 0.61 m/s. DT ranged from 120 to 268 ms with a mean (±SD) of 195.83 ± 38.53 ms, and IVRT ranged from 75 to 144 ms with a mean (±SD) of 107.91 ± 19.71 ms.

**Figure 1:**
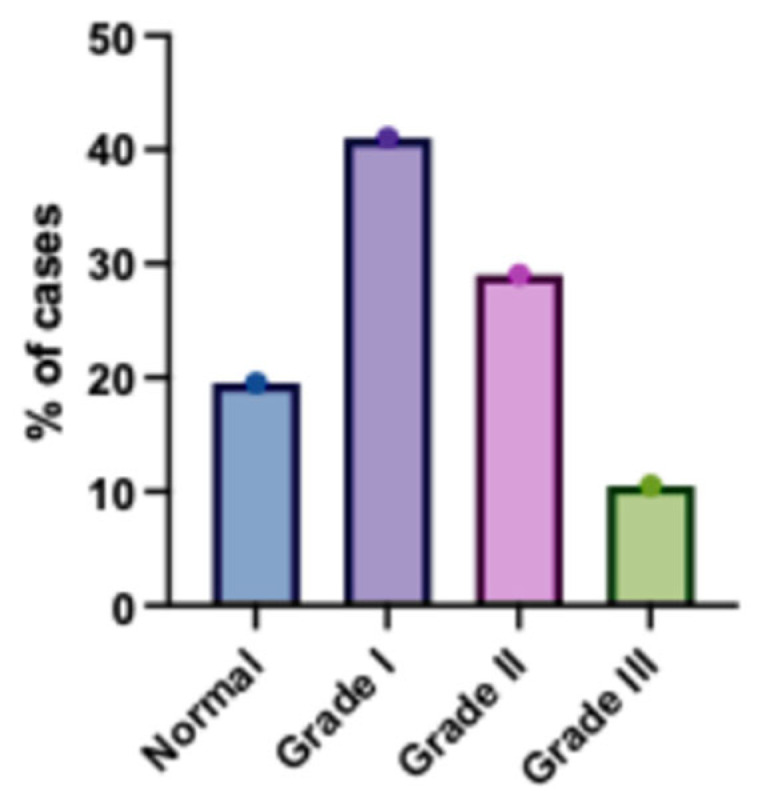
Distribution of Diastolic Dysfunction Grades Among the Studied Patients. The bar chart illustrates the prevalence of different left ventricular diastolic dysfunction grades. Grade I (impaired relaxation) was the most common, followed by Grade II (pseudonormal), while Grade III (restrictive filling) showed the lowest occurrence, indicating a predominance of milder forms of diastolic dysfunction in the cohort. Error bars represent standard deviation.

Among the studied patients, 39 (19.5%) patients had normal diastolic function, 82 (41%) patients had diastolic dysfunction grade I, 58 (29%) patients had diastolic dysfunction grade II and 21 (10.5%) patients had diastolic grade III (Figure 1).

### 3.4. CACS of the Studied Patients

**Figure 2:**
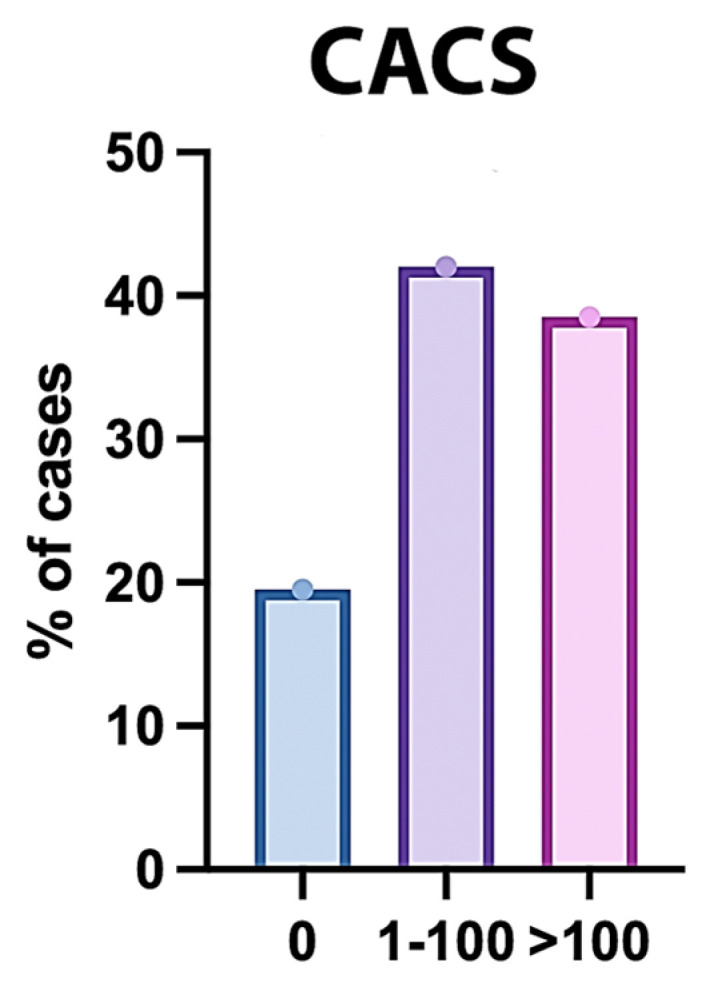
Distribution of CACS Among the Studied Patients.

The Coronary Artery Calcium Score was assessed in 200 participants. The mean (±SD) score was 143.87 ± 144.42, with a median (IQR) of 77 (16.75–286.75) and a range of 0 to 425. Regarding the distribution, 39 participants (19.5%) had a score of 0, 84 participants (42%) had scores ranging from 1 to 100, and 77 participants (38.5%) had scores greater than 100 (Figure 2).

### 3.5. Segment Involvement Score (SIS) of the Studied Patients

**Figure 3:**
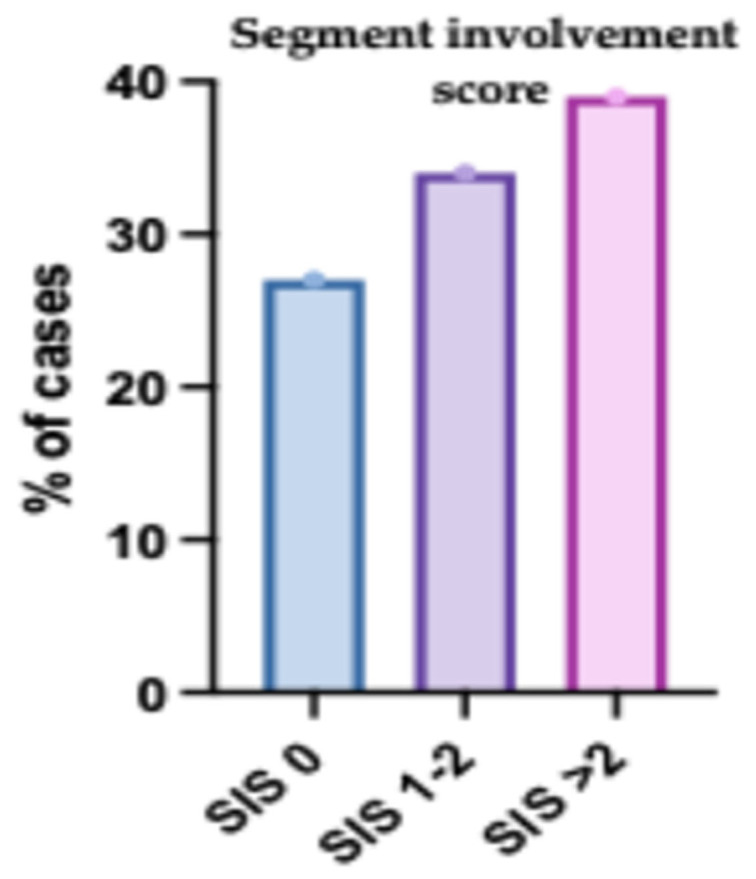
Segment Involvement Score (SIS) Distribution Among the Studied Patients. The bar chart displays the distribution of SIS values, reflecting the extent of coronary artery involvement. Patients with higher SIS were more prevalent, indicating a greater burden of atherosclerotic plaque across multiple coronary segments. Error bars represent standard deviation.

The SIS was evaluated in 200 participants. The mean (± SD) score was 2.2 ± 1.87, with a median (IQR) of 2 (0–4) and a range of 0 to 6. In terms of distribution, 54 participants (27%) had a score of 0, 68 participants (34%) had scores of 1 or 2, and 78 participants (39%) had scores greater than 2 (Figure 3).

The studied patients were classified according to the type of CAD into non-obstructive CAD in 127 (63.5%) and obstructive CAD in 73 (36.5%). Based on the type of CAD, age was significantly higher in obstructive CAD compared to non-obstructive CAD (*p* < 0.001). Sex was significantly different between obstructive CAD and non-obstructive CAD (*p* < 0.001). Both groups’ weight, height and BMI were insignificantly different (Table 4).

**Figure 4:**
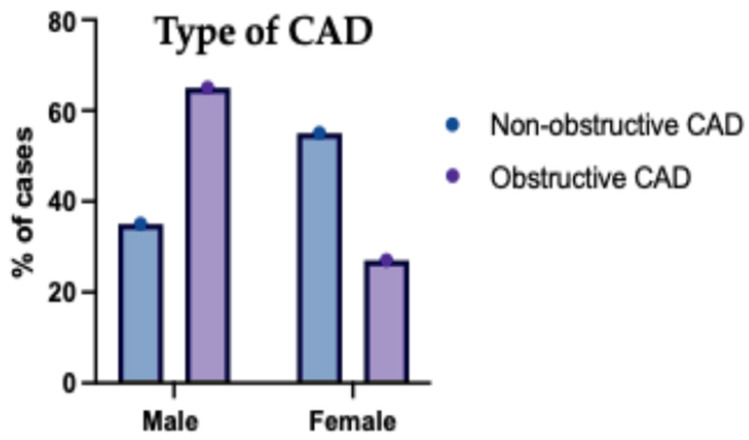
Sex distribution of the studied groups according to type of CAD. This bar chart displays the proportions of males and females within the non-obstructive and obstructive CAD cohorts. Males predominated in the obstructive CAD group, whereas females were more common in the non-obstructive group. Error bars represent standard deviation.

The gender distribution among patients categorized with non-obstructive versus obstructive (CAD) is presented in Figure 4. Within the non-obstructive CAD group, females represented a higher proportion, estimated at approximately 56%, while males constituted about 44%. Conversely, in the obstructive CAD group, males formed the majority, estimated at around 68%, with females accounting for the remaining approximately 32%. This indicates a notable difference in gender distribution between the two CAD categories presented in the study population.

Regarding the risk factors, hypertension and DM were significantly higher in obstructive CAD compared to non-obstructive CAD (*p* < 0.001, <0.001), with no significant difference between both groups regarding smoking, family history and dyslipidemia (Table 5).

A comparison of baseline laboratory findings between patients with non-obstructive CAD (*n* = 127) and obstructive CAD (*n* = 73) revealed several statistically significant differences, particularly in glycemic control and lipid profiles. Patients with obstructive CAD had significantly higher mean HbA1c levels (5.67 ± 1.67% vs. 4.98 ± 1.35%, *p* = 0.002). Furthermore, the obstructive CAD group exhibited a significantly more atherogenic lipid profile, characterized by higher mean total cholesterol (281.81 ± 44.65 vs. 198.3 ± 40.32 mg/dL, *p* < 0.001), triglycerides (212.33 ± 43.83 vs. 187.77 ± 25.59 mg/dL, *p* < 0.001), and LDL cholesterol (129.18 ± 25.06 vs. 100.7 ± 16.93 mg/dL, *p* < 0.001), along with significantly lower mean HDL cholesterol levels (35.22 ± 3.05 vs. 43.02 ± 4.72 mg/dL, *p* < 0.001). No significant differences were observed between the groups for hemoglobin, white blood cell count, platelet count, ALT, AST, creatinine, or urea levels (Table 6).

Comparing echocardiographic findings between non-obstructive (*n* = 127) and obstructive CAD (*n* = 73) groups revealed highly significant differences across nearly all parameters (*p* < 0.001). Patients with obstructive CAD exhibited significantly lower mean ejection fractions (57.45% vs. 60.32%) and larger left atrial volumes (LAVI 33.96 vs. 30.64 mL/m^2^). Furthermore, multiple indices indicated significantly worse diastolic function in the obstructive group, including higher E/e′ ratios (10.1 vs. 8.07) and a markedly higher prevalence of overall diastolic dysfunction (71.2% vs. 23.6%), particularly grades II and III (Table 7).

**Figure 5:**
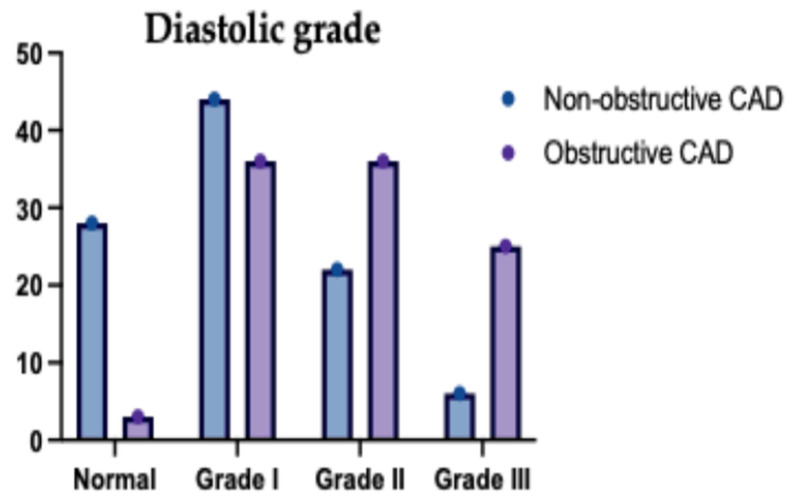
Diastolic Grade of the Studied Groups Regarding the Type of CAD. This bar chart compares the distribution of diastolic dysfunction grades (I, II, III) between patients with non-obstructive CAD and those with obstructive CAD. Grade I diastolic dysfunction was more prevalent among patients with non-obstructive CAD, whereas Grade III predominated among those with obstructive CAD, indicating that obstructive lesions are associated with more severe diastolic impairment. Grade II dysfunction occurred in both groups but was more frequent among patients with obstructive disease. These findings suggest that the presence of obstructive coronary lesions is associated with a progressive worsening of diastolic function.

**Figure 6:**
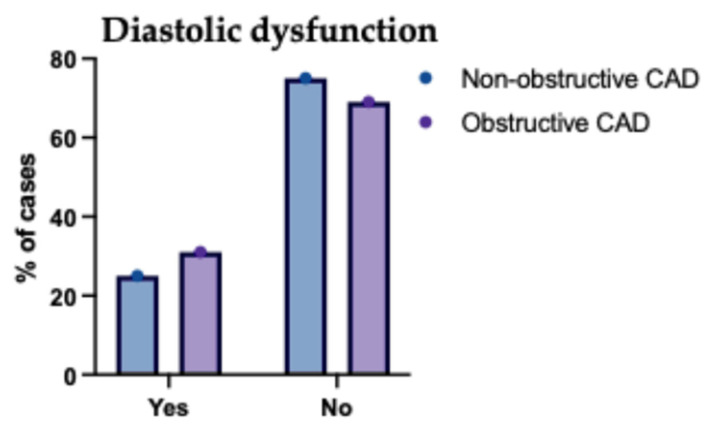
Presence of Diastolic Dysfunction in the Studied Groups Regarding the Type of CAD. This figure illustrates the proportion of patients with normal diastolic function versus any degree of diastolic dysfunction in the non-obstructive and obstructive CAD groups. Patients with obstructive CAD had a markedly higher prevalence of diastolic dysfunction compared to those with non-obstructive disease. Conversely, normal diastolic function was more common among patients with non-obstructive CAD. These data support a strong association between obstructive coronary lesions and impaired diastolic function and underscore the importance of early cardiac function evaluation in patients with significant obstructive diseases.

The relationship between the extent of (CAD) and left ventricular diastolic function in the studied patient groups is illustrated in Figure 5 and Figure 6. As shown in Figure 5, there is a clear trend of increasing severity of diastolic dysfunction (Grades II and III) with greater CAD burden, particularly in patients with two- and multivessel disease. Figure 6 further reinforces this association by demonstrating a higher prevalence of diastolic dysfunction among those with more extensive CAD involvement, while patients with single-vessel disease more frequently retained normal diastolic function. These findings suggest a strong correlation between CAD complexity and the deterioration of myocardial relaxation, underscoring the importance of comprehensive cardiac assessment in patients with advanced coronary pathology.

**Figure 7:**
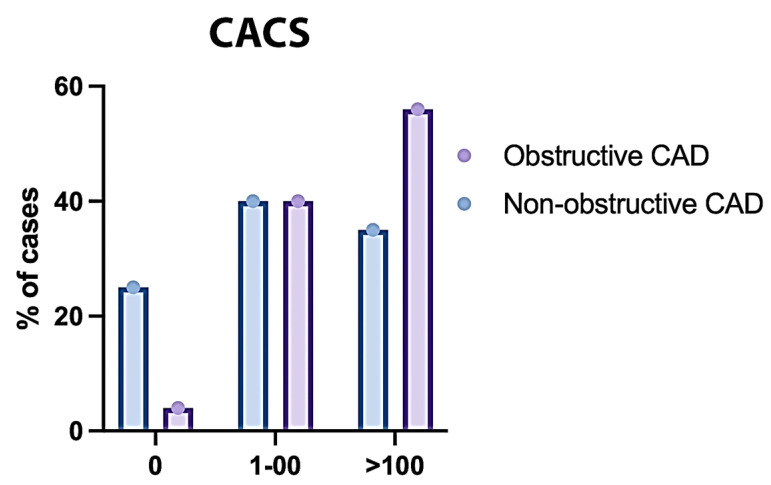
CACS of the Studied Groups Regarding the Type (CAD).

The mean CACS was significantly higher in obstructive CAD compared to non-obstructive CAD (*p* < 0.001). CACS was significantly different between both groups (*p* < 0.001); CACS > 100 was significantly higher in obstructive CAD compared to non-obstructive CAD (58.9% vs. 26.77%) (Table 8 and Figure 7).

**Figure 8:**
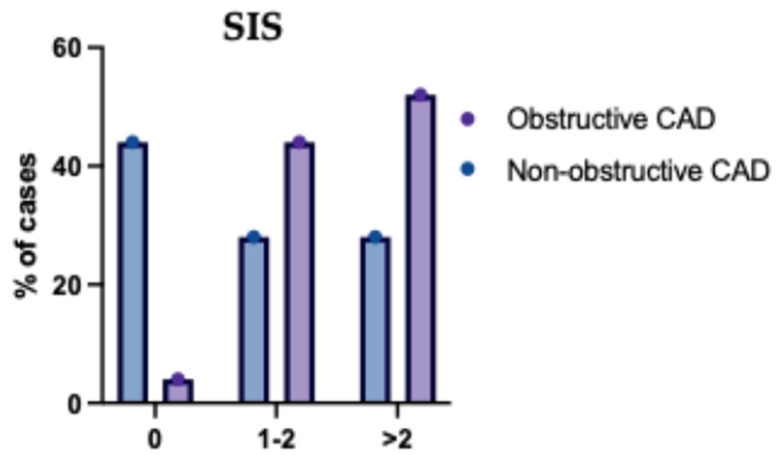
Segment Involvement Score (SIS) of the Studied Groups Regarding the Type of (CAD). This bar chart displays the distribution of SIS categories across single-, two-, and multivessel disease groups. Patients with multivessel CAD exhibited significantly higher SIS values, indicating more extensive atherosclerotic involvement. In contrast, lower SIS were predominantly observed in the single-vessel disease group. The results support a positive correlation between the number of affected vessels and overall plaque burden. Error bars represent standard deviation.

The mean SIS was significantly higher in obstructive CAD compared to non-obstructive CAD (*p* < 0.001). SIS was significantly different between both groups (*p* < 0.001); SIS > 2 was significantly higher in obstructive CAD compared to non-obstructive CAD (57.53% vs. 28.35%) (Table 9 and Figure 8).

There was a significant relation between the incidence of diastolic dysfunction, as age was significantly higher in the diastolic dysfunction group compared to the non-diastolic dysfunction group (*p* = 0.020). There was an insignificant relation between the incidence of diastolic dysfunction and other baseline characteristics (sex, weight, height and BMI) (Table 10).

Positive family history, hypertension and DM were significantly higher in the diastolic dysfunction group compared to the non-diastolic dysfunction group (*p* < 0.05). There was an insignificant relation between diastolic dysfunction and smoking and dyslipidemia (Table 11).

Patients with diastolic dysfunction had significantly higher total cholesterol, LDL (*p* < 0.05), and significantly lower Hb concentration, WBCs count, and HDL compared to those without diastolic dysfunction (*p* = 0.020, 0.050), with no significant difference between both groups regarding the other laboratory investigations (platelet count, HbA1c, ALT, AST, TG, serum creatinine and urea) (Table 12).

There was a significant relationship between diastolic dysfunction and echocardiographic findings, except for TR velocity and LVH, which were insignificantly different between diastolic and non-diastolic dysfunction groups. EF, septal e′, lateral e′, DT and IVRT were significantly lower in the diastolic and non-diastolic dysfunction groups (*p* < 0.05). LAVI, E/e′, E/A and both diastolic function grade II and grade III were significantly higher in the diastolic dysfunction group compared to the non-diastolic dysfunction group (*p* < 0.05) (Table 13).

### 3.6. The Relationship Between Diastolic Dysfunction and CACS

The relationship between diastolic dysfunction and CACS was examined by comparing patients with diastolic dysfunction (*n* = 82) to those without (*n* = 118). A highly significant difference was observed in mean CACS, with patients experiencing diastolic dysfunction reporting substantially higher scores (mean ± SD: 236.33 ± 133.12 vs. 79.61 ± 114.23, *p* < 0.001). The distribution of scores also differed significantly (*p* < 0.001); notably, no patients with diastolic dysfunction had a score of 0 (compared to 33.05% of those without), while a much larger proportion had scores greater than 100 (69.51% vs. 16.95%). There was a significant relation between diastolic dysfunction and CACS. The mean CACS was significantly higher in the diastolic dysfunction group compared to the non-diastolic dysfunction group (*p* < 0.001). Also, patients with CACS >100 were significantly higher in the diastolic dysfunction group compared to non-diastolic dysfunction group (*p* < 0.001).

### 3.7. Relation Between Diastolic Dysfunction and Segment Involvement Score (SIS)

Similarly, the SIS was compared between the two groups. Patients with diastolic dysfunction (*n* = 82) had significantly higher mean SIS compared to those without (*n* = 118) (mean ± SD: 3.41 ± 1.48 vs. 1.35 ± 1.63, *p* < 0.001). The distribution across SIS categories also showed a significant difference (*p* < 0.001), with fewer patients in the diastolic dysfunction group having a score of 0 (13.41% vs. 52.44%) and a substantially higher proportion having scores greater than 2 (69.51% vs. 25.61%). There was a significant association between diastolic dysfunction and SIS. The mean SIS was significantly higher in the diastolic dysfunction group compared to the non-diastolic dysfunction group (*p* < 0.001). Also, patients with SIS > 2 were significantly higher in the diastolic dysfunction group compared to non-diastolic dysfunction group (*p* < 0.001). Furthermore, a strong positive correlation was found between the Segment Involvement Score (SIS) and the severity of diastolic function grades (r = 0.809, *p* < 0.001).

**Figure 9:**
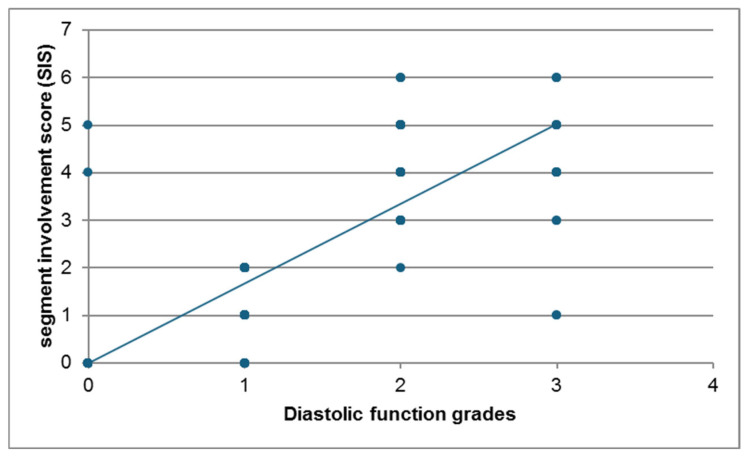
Scatter Plot Demonstrating the Positive Correlation Between Segment Involvement Score (SIS) and Diastolic Function Grades. The scatter plot illustrates a positive linear relationship between SIS and diastolic dysfunction grades, indicating that higher diastolic impairment is associated with greater coronary segment involvement. This trend suggests a potential link between the extent of coronary artery disease and worsening left ventricular diastolic function.

There was a strong positive correlation between diastolic dysfunction grades (Echocardiography) and segment involvement score (SIS) (r = 0.809 and *p* value < 0.001). The scatter plot (Figure 9) illustrates the relationship between the Segment Involvement Score (SIS) and diastolic function grades for the study participants. A clear positive association is visible between the two variables shown. Higher SIS tend to correspond with more severe grades of diastolic dysfunction. While there is some scatter, the overall trend indicates a strong positive correlation.

### 3.8. Baseline Demographic Characteristics

Baseline demographic characteristics were compared across the three groups defined by CACS: score 0 (*n* = 39), score 1–100 (*n* = 84), and score > 100 (*n* = 77). No statistically significant differences were observed among these groups regarding sex distribution, mean weight, mean height, or mean Body Mass Index (BMI) (all *p* > 0.4). A non-significant trend towards slightly older age was noted in patients with higher CACS (*p* = 0.063) (Table 14).

There was a significant relation between CACS and family history (*p* = 0.034), positive family history was significantly higher in patients with CACS > 100. There was an insignificant relation between CACS and the other risk factors (smoking, hypertension, DM and dyslipidemia) (Table 15).

Analysis of laboratory parameters across CACS groups (0, *n* = 39; 1–100, *n* = 84; >100, *n* = 77) revealed significant differences primarily in the lipid profile. Patients with higher CACS (>100) exhibited significantly higher mean total cholesterol (*p* = 0.004) and LDL cholesterol (*p* = 0.018) compared to those with a score of 0. Conversely, mean HDL cholesterol was significantly lower in the >100 group compared to both the 0 group (*p* = 0.001) and the 1–100 group (*p* = 0.046). No significant differences were found for other measured parameters, including HbA1c and triglycerides (Table 16).

Echocardiographic parameters were also compared across CACS groups. Significant differences were primarily observed in diastolic function indices and left atrial size (LAVI, *p* = 0.007). Patients with higher CACS (>100) generally showed indicators of worse diastolic function, including significantly lower septal e′ (*p* = 0.017) and lateral e′ velocities (*p* = 0.014), higher E/e′ ratios (*p* = 0.009), higher TR velocity (*p* = 0.002), shorter DT (*p* = 0.010), and shorter IVRT (*p* < 0.001), particularly when compared to the CACS 0 group. Correspondingly, the distribution of diastolic function grades differed significantly (P<0.001), with worse grades more prevalent in higher CACS groups. No significant differences were found in ejection fraction or LVH prevalence (Table 17).

Furthermore, the Segment Involvement Score (SIS) was compared across the CACS groups, revealing a strong and highly significant association (Overall *p* < 0.001). Mean SIS progressively increased with higher CACS categories (CACS 0: 0.28 ± 1.04; CACS 1–100: 1.25 ± 0.77; CACS > 100: 4.25 ± 0.9), with all pairwise comparisons being statistically significant (*p* < 0.001). The distribution of SIS also differed significantly (*p* < 0.001); virtually all patients with CACS 0 had an SIS of 0, most patients with CACS 1–100 had an SIS of 1–2, and the vast majority of patients with CACS > 100 had an SIS greater than 2 (Table 18).

In the univariate analysis, age, diabetes mellitus (DM), hypertension (HTN) and male sex were significant risk factors for diastolic dysfunction, as were E/E′, lower ejection fraction (EF), left atrial volume index (LAVI) and tricuspid regurgitation (TR) velocity on echocardiography. Higher CACS, higher SIS and the presence of obstructive CAD were also significantly associated with diastolic dysfunction. After multivariable adjustment for age and major risk factors, obstructive CAD remained independently associated with diastolic dysfunction in conjunction with higher SIS, elevated CACS, increased E/E′ and larger LAVI. By contrast, TR velocity and EF did not show a significant relationship with the severity of CAD obstruction.

## 4. Discussion

Atherosclerotic cardiovascular disease often develops silently over decades, representing a highly prevalent subclinical condition in adults. While non-obstructive plaques may not initially cause ischemia, they can induce functional vascular changes linked to endothelial dysfunction and impaired nitric oxide production [15]. Concurrently, left ventricular (LV) diastolic dysfunction (DD), often preceding systolic impairment, is increasingly recognized as a significant cause of heart failure with preserved ejection fraction (HFpEF) and a predictor of adverse outcomes, including overt heart failure and mortality [16]. Conditions like aging, hypertension, and (CAD) can exacerbate LV relaxation abnormalities and increase filling pressures, potentially leading to HFpEF [16]. Given the prognostic importance of DD, its early detection via noninvasive methods like echocardiography, particularly using tissue Doppler imaging to overcome the limitations of traditional measures, is crucial [17].

Despite the established impact of overt ischemic heart disease, the relationship between the severity of CAD, particularly in its earlier or non-obstructive stages, and the degree of LV diastolic dysfunction has remained incompletely understood, with limited and sometimes contradictory evidence [18,19,20]. Therefore, this study aimed to determine the correlation between CAD severity, comprehensively assessed by (CCTA), and the grade of LV diastolic dysfunction evaluated by (TTE) in patients with an intermediate pretest probability of CAD.

A central finding of this study is the strong and highly significant positive correlation between the severity of CAD, measured by CCTA indices, and the degree of LV diastolic dysfunction. Patients with obstructive CAD (higher CAD-RADS scores) consistently demonstrated echocardiographic parameters indicative of worse diastolic function compared to those with non-obstructive disease. This included significantly larger left atrial volume index (LAVI), lower mitral annular velocities (septal and lateral e’), higher E/e’ ratios, higher E/A ratios (though the overall group difference for E/A was borderline, *p* = 0.075, the trend aligned), higher tricuspid regurgitation (TR) velocities, and shorter deceleration times (DT) and isovolumetric relaxation times (IVRT) (all *p* < 0.01). These findings strongly support a pathophysiological link where increasing coronary atherosclerotic burden compromises myocardial relaxation and compliance, leading to elevated LV filling pressures. Our results align closely with those of [14], who also utilized CCTA and TTE and found significantly worse diastolic parameters (lower DT/IVRT, higher E/e′, E/A, LAVI, TR velocity) and a higher prevalence of DD (particularly advanced grades) in patients with obstructive CAD compared to non-obstructive CAD.

Furthermore, we observed a robust correlation between CT-derived plaque burden scores and diastolic function. Both the CACS and the SIS showed highly significant positive associations with the severity of diastolic dysfunction (*p* < 0.001 for both mean scores and distribution across categories). Patients with higher CACS (>100) and SIS (>2) scores exhibited markedly worse diastolic function parameters and a higher prevalence of moderate-to-severe DD compared to those with lower scores. This reinforces the concept that not just obstructive stenosis but the overall extent of coronary atherosclerosis contributes to diastolic impairment. This is consistent with [21], who found that higher CACS and SIS were associated with a greater prevalence of DD (specifically grades >2), and also aligns with prognostic studies showing that higher SIS predicts adverse events, even among those with obstructive CAD [22,23,24]. Our findings contrast with some earlier studies suggesting an inverse or absent relationship between subclinical atherosclerosis markers (like carotid intima–media thickness or CACS in asymptomatic populations) and DD [19,25]. These discrepancies may stem from differences in the methods used to assess atherosclerosis (CIMT or CACS vs. comprehensive CCTA, including non-calcified plaque via SIS/CAD-RADS), the populations studied (healthy/asymptomatic vs. symptomatic intermediate-risk), and the sensitivity of echocardiographic techniques employed. Critically, our study, utilizing detailed CCTA and contemporary echocardiographic guidelines in a relevant clinical population, provides substantial evidence supporting a direct, graded relationship between coronary atherosclerotic burden and the severity of diastolic dysfunction.

Chronic myocardial ischemia due to atherosclerotic narrowing can lead to repetitive episodes of subendocardial hypoperfusion. Over time this causes impaired calcium handling, delayed myocardial relaxation and progressive stiffening of the ventricle. In addition, microvascular dysfunction resulting from endothelial injury and impaired nitric oxide bioavailability diminishes coronary flow reserve and exacerbates ischemic injury even in the absence of large–vessel obstruction. Atherosclerosis is also a systemic inflammatory disease; circulating inflammatory cytokines and oxidative stress promote interstitial myocardial fibrosis, which increases ventricular stiffness and further impedes diastolic relaxation. Finally, accumulation of diffuse myocardial fibrosis—as occurs with chronic hypertension, diabetes and aging—can compound the effects of coronary atherosclerosis on diastolic function. These mechanisms collectively explain why greater plaque burden and obstructive lesions correlate with more severe diastolic dysfunction in our cohort.

Consistent with established epidemiology, patients with obstructive CAD in our cohort were significantly older than those with non-obstructive CAD, although age did not show a statistically significant association with CACS categories (*p* = 0.063). This aligns with large registry data showing a higher prevalence of obstructive CAD in older symptomatic patients referred for CCTA [26]. We also observed a significantly higher proportion of males in the obstructive CAD group (*p* < 0.001). This contrasts with [27], who found similar frequencies of obstructive CAD in men and women evaluated with CCTA/FFR for type 2 myocardial infarction. This discrepancy might be explained by population differences (our broader intermediate-risk CAD population vs. their specific type 2 MI cohort) and potential variations in underlying pathophysiology (atherosclerotic obstruction vs. supply–demand mismatch), cardiovascular risk profiles, and hormonal influences between the study groups [27].

Hypertension and diabetes mellitus were significantly more prevalent in patients with obstructive CAD (*p* < 0.001) and also in those with diastolic dysfunction (*p* < 0.001) in our study. This confirms the well-known association of these risk factors with both advanced atherosclerosis and impaired diastolic function [28]. The strong link between these conditions and DD is further supported by [29], who demonstrated in a community cohort that hypertension and diabetes independently predicted worse diastolic function (higher E/e’), with their coexistence conferring an additive negative impact, potentially explaining the increased risk of heart failure in such patients [30,31]. While previous studies examining these relationships used older diagnostic criteria or lacked advanced echo techniques [32,33], our findings, adjusted implicitly through group comparisons, reinforce the crucial role of hypertension and diabetes in the development of both obstructive CAD and diastolic dysfunction.

Glycemic control, reflected by HbA1c, was significantly worse in the obstructive CAD group compared to the non-obstructive group (*p* = 0.002), consistent with studies linking higher HbA1c levels to increased severity and complexity of coronary lesions, even in non-diabetic individuals [34,35]. However, HbA1c levels did not significantly differ across CACS categories in our analysis (*p* = 0.903), suggesting that while poor glycemic control is associated with obstructive disease, its relationship with the overall calcified plaque burden might be less direct or influenced by other factors within this specific population.

Lipid profiles also showed significant associations. Patients with obstructive CAD had significantly higher total cholesterol, LDL, triglycerides, and lower HDL than the non-obstructive group (all *p* < 0.001). Similarly, worse lipid profiles (higher total cholesterol and LDL, lower HDL) were significantly associated with higher CACS (*p* = 0.005, *p* = 0.022, *p* = 0.001, respectively), aligning with findings by [36] who reported correlations between lipids and CACS. Furthermore, patients with diastolic dysfunction exhibited significantly higher total cholesterol and LDL levels compared to those without DD. This association between dyslipidemia and DD, particularly in diabetic populations, has been noted previously [37]. Our finding of higher LDL in the obstructive CAD group contrasts with [38], who found higher LDL in non-obstructive patients post-angiography. As suggested by Wilkinson et al., this discrepancy likely reflects differences in study design and timing; their post-procedural assessment may capture the effects of aggressive lipid-lowering initiated after diagnosing obstructive disease, whereas our baseline assessment reflects the untreated state.

Notably, although Table 10 indicated no significant association between patient sex and the presence of diastolic dysfunction (*p* = 0.210), the univariate regression analysis presented in Table 19 identified male sex as a significant risk factor (*p* = 0.016). This apparent inconsistency is largely methodological: the chi-square comparison in Table 10 examines crude differences in proportions, whereas the regression model evaluates the odds of diastolic dysfunction while accounting for continuous and categorical covariates. When the odds ratios were modeled, male sex emerged as an independent predictor (Table 20).

While the primary focus was diastolic function, we observed that mean ejection fraction (EF), although within the normal range for both groups, was significantly lower in patients with obstructive CAD compared to non-obstructive CAD (57.45% vs. 60.32%, *p* < 0.001). This aligns with [39] and suggests that even in patients largely classified as having preserved EF, obstructive CAD may be associated with subtle systolic impairment. However, EF did not differ significantly across CACS (*p* = 0.780) or between those with and without diastolic dysfunction, consistent with the concept of HFpEF, where significant diastolic abnormalities can exist despite relatively normal EF [21,40].

This study underscores the strong association between the severity and extent of coronary atherosclerosis, assessed by CCTA, and the presence and severity of LV diastolic dysfunction, evaluated by contemporary echocardiography. Identifying DD in patients with CAD, even non-obstructive disease, is clinically relevant as it signifies impaired cardiac mechanics and carries prognostic weight. The consistent link across various CCTA scores (CAD-RADS, CACS, SIS) and multiple echocardiographic parameters strengthens the evidence for this relationship in an intermediate-risk population. These findings highlight the potential utility of integrating both CCTA and detailed echocardiographic assessments for comprehensive cardiovascular risk stratification. Recognizing the coexistence of significant CAD and DD may prompt more aggressive management of shared risk factors like hypertension, diabetes, and dyslipidemia, potentially mitigating the progression to HFpEF and improving patient outcomes.

The finding that higher SIS and CACS values were strongly associated with diastolic impairment even among patients without obstructive stenoses has important clinical implications. Total plaque burden reflects the overall inflammatory and ischemic milieu of the coronary vasculature and appears to be a key determinant of diastolic dysfunction independent of flow-limiting lesions. Accordingly, patients with extensive non-obstructive plaque should not be considered benign; instead, they warrant careful longitudinal follow–up and aggressive control of cardiovascular risk factors to mitigate progression to heart failure. Early recognition and management of diastolic dysfunction in this population may allow clinicians to intervene before irreversible ventricular remodeling occurs

Strengths of this study include the use of comprehensive CCTA for detailed CAD assessment (including plaque burden via SIS and CACS, and stenosis severity via CAD-RADS) and contemporary ASE/EAE guideline-based echocardiography for diastolic function grading. The recruitment of a relevant clinical population with intermediate pretest probability enhances the generalizability of the findings to patients commonly undergoing non-invasive cardiac imaging.

## 5. Conclusions

In conclusion, this study demonstrates a significant and graded positive correlation between the severity and extent of coronary artery disease, assessed by CCTA, and the presence and severity of left ventricular diastolic dysfunction, assessed by echocardiography, in patients with intermediate pretest probability for CAD. Higher CAD-RADS classifications, CACS, and SIS were consistently associated with worse diastolic function parameters and higher grades of diastolic dysfunction. Key cardiovascular risk factors, notably hypertension and diabetes, along with dyslipidemia, were associated with both obstructive CAD and diastolic dysfunction. These findings emphasize the interconnectedness of coronary atherosclerosis and diastolic impairment and highlight the importance of comprehensive cardiovascular assessment for risk stratification and management. Future longitudinal studies are warranted to confirm these associations and explore the impact of therapeutic interventions targeting CAD and risk factors on diastolic function progression. This study is limited by its small sample size, single-center design, and short follow-up period, which may affect the generalizability and long-term applicability of the findings. Future research should include larger, multicenter cohorts with extended follow-up to evaluate disease progression and treatment outcomes better.

### Study Limitations

This study has several limitations that should be considered when interpreting the findings. The cross–sectional design limits causal inference, allowing only associations to be described. Data were collected from a single center and the moderate sample size, although adequate for detecting the main effects, may reduce the generalizability of the results and limit statistical power for subgroup analyses. While CCTA and TTE were performed within a maximum interval of one year, they were not simultaneous; the mean (±SD) interval between examinations was 6.2 ± 3.0 months with a median of 6 months (IQR 3–9 months), leaving room for interim clinical changes that might influence the relationship between coronary anatomy and diastolic function. Our imaging protocol assessed anatomical stenosis without functional ischemia testing (e.g., fractional flow reserve or stress echocardiography) and did not include longitudinal follow-up, precluding assessment of the hemodynamic significance of lesions or evaluation of disease progression. Although we adjusted for major cardiovascular risk factors, including hypertension and diabetes as covariates in multivariate analyses, residual confounding from unmeasured variables—such as genetic factors, lifestyle habits or medication adherence—cannot be completely ruled out. Finally, the absence of an external validation cohort and the complex, heterogeneous pathophysiology of diastolic dysfunction limit extrapolation of these findings to other populations. Future multicenter longitudinal studies using comprehensive functional and anatomic assessments are needed to clarify causal relationships and verify our results.

## Figures and Tables

**Table 1:** Baseline characteristics of the studied patients.

Parameter		Total (*n* = 200)
Age (Years)	Mean ± SD	54.27 ± 8.98
	Range	40–73
Sex	Male	104 (52%)
	Female	96 (48%)
Weight (Kg)	Mean ± SD	74.83 ± 12.45
	Range	55–95
Height (m)	Mean ± SD	1.68 ± 0.05
	Range	1.58–1.75
BMI (Kg/m^2^)	Mean ± SD	26.73 ± 4.73
	Range	18.29–36.65

**Table 2:** Complete blood count, HbA1C, Liver Enzymes, Lipid Profile, and Kidney Functions of the studied patients.

		Total (*n* = 200)
Hb (g/dL)	Mean ± SD	12.33 ± 1.14
	Range	10.2–14.2
WBCs (×10^9^/L)	Mean ± SD	8.14 ± 1.99
	Range	4.5–11.5
Platelets (×10^9^/L)	Mean ± SD	281.25 ± 43.58
	Range	201–349
HbA1c (%)	Mean ± SD	5.23±1.51
	Range	3.5–8.5
ALT (U/L)	Mean ± SD	31.98 ± 8.35
	Range	18–45
AST (U/L)	Mean ± SD	33.05 ± 7.45
	Range	20–45
Cholesterol (mg/dL)	Mean ± SD	228.78 ± 58.1
	Range	130–349
TG (mg/dL)	Mean ± SD	196.74 ± 35.36
	Range	141–289
LDL (mg/dL)	Mean ± SD	111.1 ±24.45
	Range	70–170
HDL (mg/dL)	Mean ± SD	40.17 ± 5.63
	Range	30–50
Creatinine (mg/dL)	Mean ± SD	1.03 ± 0.14
	Range	0.8–1.2
Urea (mg/dL)	Mean ± SD	44.38 ± 14.92
	Range	20–70

**Table 3:** Echocardiography of the studied patients.

		Total (*n* = 200)
EF (%)	Mean ± SD	59.28 ± 5.03
	Range	49–69
LAVI (mL/m^2^)	Mean ± SD	31.85 ± 3.74
	Range	26–43
Septal e′ (m/s)	Mean ± SD	0.83 ± 0.17
	Range	0.5–1.1
Lateral e′ (m/s)	Mean ± SD	0.12 ± 0.05
	Range	0.03–0.25
E/e′	Mean ± SD	8.81 ± 1.85
	Range	6–13.6
E/A	Mean ± SD	1.39 ± 0.44
	Range	0.71–2.55
TR velocity (m/s)	Mean ± SD	1.85 ± 0.61
	Range	0.8–3.1
DT (ms)	Mean ± SD	195.83 ± 38.53
	Range	120–268
IVRT (ms)	Mean ± SD	107.91 ± 19.71
	Range	75–144
Diastolic function grade	Normal	39 (19.5%)
	Grade I	82 (41%)
	Grade II	58 (29%)
	Grade III	21 (10.5%)
Diastolic dysfunction		82 (41%)
LVH		37 (18.5%)

**Table 4:** Baseline characteristics of the studied groups regarding the type of CAD.

		Non-Obstructive CAD (*n* = 127)	Obstructive CAD (*n* = 73)	*p* Value
Age (years)	Mean ± SD	52.18 ± 8.34	57.9 ± 8.94	**<0.001**
	Range	40–65	45–73	
Sex	Male	53 (41.73%)	51 (69.86%)	**<0.001**
	Female	74 (58.27%)	22 (30.14%)	
Weight (kg)	Mean ± SD	73.92 ± 12.53	76.41 ± 12.22	0.173
	Range	55–95	55–94	
Height (m)	Mean ± SD	1.67 ± 0.05	1.68 ± 0.05	0.201
	Range	1.59–1.75	1.58–1.75	
BMI (kg/m^2^)	Mean ± SD	26.52 ± 4.81	27.09 ± 4.6	0.412
	Range	18.29–36.65	18.38–35.88	

**Table 5:** Risk factors of the studied groups regarding the type of CAD.

	Non-Obstructive CAD (*n* = 127)	Obstructive CAD (*n* = 73)	*p* Value
Smoking	29 (22.83%)	23 (31.51%)	0.178
Family history	18 (14.17%)	25 (34.25%)	0.001
Hypertension	38 (29.92%)	42 (57.53%)	0.001
DM	24 (18.9%)	33 (45.21%)	0.001
Dyslipidaemia	40 (31.5%)	26 (35.62%)	0.551

**Table 6:** Complete blood count, HbA1C, Liver Enzymes, Lipid Profile, Kidney Functions of the studied patients regarding the type of CAD.

		Non-Obstructive CAD (*n* = 127)	Obstructive CAD (*n* = 73)	*p* Value
Hb (g/dL)	Mean ± SD	12.35 ± 1.11	12.31 ± 1.19	0.832
	Range	10.2–14.2	10.2–14.2	
WBCs (×10^9^/L)	Mean ± SD	8.23 ± 1.88	7.98 ± 2.17	0.392
	Range	4.5–11.5	4.5–11.5	
Platelets (×10^9^/L)	Mean ± SD	280.62 ± 44.66	282.34 ± 41.92	0.788
	Range	204–348	201–349	
HbA1c (%)	Mean ± SD	4.98±1.35	5.67±1.67	0.002
	Range	3.5–8.5	3.5–8.5	
ALT (U/L)	Mean ± SD	31.65 ± 8.19	32.53 ± 8.66	0.474
	Range	18–45	18–45	
AST (U/L)	Mean ± SD	32.77 ± 7.56	33.52 ± 7.3	0.495
	Range	20–45	21–45	
Cholesterol (mg/dL)	Mean ± SD	198.3 ± 40.32	281.81 ± 44.65	<0.001
	Range	130–259	203–349	
TG (mg/dL)	Mean ± SD	187.77 ± 25.59	212.33 ± 43.83	<0.001
	Range	141–229	141–289	
LDL (mg/dL)	Mean ± SD	100.7 ± 16.93	129.18 ± 25.06	<0.001
	Range	70–130	82–170	
HDL (mg/dL)	Mean ± SD	43.02 ± 4.72	35.22 ± 3.05	<0.001
	Range	35–50	30–40	
Creatinine (mg/dL)	Mean ± SD	1.02 ± 0.14	1.05 ± 0.13	0.167
	Range	0.8–1.2	0.8–1.2	
Urea (mg/dL)	Mean ± SD	44.06 ± 14.18	44.95 ± 16.21	0.685
	Range	20–70	20–70	

**Table 7:** Echocardiography of the studied groups regarding the type of CAD.

		Non-Obstructive CAD (*n* = 127)	Obstructive CAD (*n* = 73)	*p* Value
EF (%)	Mean ± SD	60.32 ± 4.73	57.45 ± 5.04	<0.001
	Range	53–69	49–65	
LAVI (mL/m^2^)	Mean ± SD	30.64 ± 2.46	33.96 ± 4.57	<0.001
	Range	26–34	27–43	
Septal e′ (m/s)	Mean ± SD	0.91 ± 0.14	0.7 ± 0.14	<0.001
	Range	0.7–1.1	0.5–0.9	
Lateral e′ (m/s)	Mean ± SD	0.13 ± 0.07	0.1 ± 0.01	<0.001
	Range	0.03–0.25	0.08–0.12	
E/e′	Mean ± SD	8.07 ± 1.18	10.1 ± 2.08	<0.001
	Range	6–10.2	6.5–13.6	
E/A	Mean ± SD	1.25 ± 0.33	1.64 ± 0.5	<0.001
	Range	0.71–1.81	0.74–2.55	
TR velocity (m/s)	Mean ± SD	1.74 ± 0.55	2.05 ± 0.65	<0.001
	Range	0.8–2.6	1–3.1	
DT (ms)	Mean ± SD	213.67 ± 30.89	164.79 ± 29.92	<0.001
	Range	163–268	120–215	
IVRT (ms)	Mean ± SD	118.87 ± 15.69	88.84 ± 7.92	<0.001
	Range	90–144	75–100	
Diastolic function grade	Normal	37 (29.13%)	2 (2.74%)	<0.001
	Grade I	54 (42.52%)	28 (38.36%)	
	Grade II	30 (23.62%)	28 (38.36%)	
	Grade III	6 (4.72%)	15 (20.55%)	
Diastolic dysfunction		30 (23.62%)	52 (71.23%)	<0.001
LVH		19 (14.96%)	18 (24.66%)	0.089

**Table 8:** CACS of the studied groups regarding the type of CAD.

		Non-Obstructive CAD (*n* = 127)	Obstructive CAD (*n* = 73)	*p* Value
CACS	Mean ± SD	103.56 ± 127.21	213.99 ± 146.54	<0.001
	Range	0–420	0–425	
	Median (IQR)	51 (0–204.5)	239 (58–332)	
	0	37 (29.13%)	2 (2.74%)	<0.001
	1–100	56 (44.09%)	28 (38.36%)	
	>100	34 (26.77%)	43 (58.9%)	

**Table 9:** Segment involvement score (SIS) of the studied groups regarding the type of CAD.

		Non-Obstructive CAD (*n* = 127)	Obstructive CAD (*n* = 73)	*p* Value
SIS	Mean ± SD	1.62 ± 1.76	3.19 ± 1.63	<0.001
	Range	0–5	1–6	
	Median (IQR)	1 (0–3)	3 (2–5)	
	0	54 (42.52%)	0 (0%)	<0.001
	1–2	37 (29.13%)	31 (42.47%)	
	>2	36 (28.35%)	42 (57.53%)	

**Table 10:** Relation between diastolic dysfunction and baseline characteristics.

		Diastolic Dysfunction		*p* Value
		Yes (*n* = 82)	No (*n* = 118)	
Age (years)		56.06 ± 9.13	53.03 ± 8.7	0.020
Sex	Male	47 (57.3%)	57 (48.3%)	0.210
	Female	35 (42.7%)	61 (51.7%)	
Weight (kg)		76.18 ± 13.19	73.89 ± 11.87	0.210
Height (m)		1.68 ± 0.05	1.67 ± 0.05	0.297
BMI (kg/m^2^)		27.07 ± 4.96	26.49 ± 4.58	0.405

**Table 11:** Relation between diastolic dysfunction and risk factors.

	Diastolic Dysfunction		*p* Value
	Yes (*n* = 82)	No (*n* = 118)	
Smoking	25 (30.5%)	27 (22.9%)	0.228
Family history	25 (30.5%)	18 (15.3%)	0.010
Hypertension	41 (50%)	39 (33.1%)	0.016
DM	31 (37.8%)	26 (22%)	0.015
Dyslipidemia	29 (35.4%)	37 (31.4%)	0.553

**Table 12:** Relation between diastolic dysfunction and laboratory investigations.

	Diastolic Dysfunction		*p* Value
	Yes (*n* = 82)	No (*n* = 118)	
Hb (g/dL)	12.26 ± 1.13	12.39 ± 1.14	0.020
WBCs (×10^9^/L)	7.8 ± 2.06	8.37 ± 1.91	0.050
Platelets (×10^9^/L)	281.33 ± 40.68	281.19 ± 45.66	0.983
HbA1c (%)	5.47 ± 1.59	5.07 ± 1.43	0.064
ALT (U/L)	32.43 ± 8.94	31.66 ± 7.94	0.525
AST (U/L)	32.9 ± 7.39	33.14 ± 7.53	0.822
Cholesterol (mg/dL)	254.46 ± 60.97	210.93 ± 48.82	<0.001
TG (mg/dL)	202.24 ± 38.53	192.91 ± 32.59	0.075
LDL (mg/dL)	118.06 ± 25.5	106.25 ± 22.55	<0.001
HDL (mg/dL)	37.95 ± 5.53	41.71 ± 5.18	<0.001
Creatinine (mg/dL)	1.05 ± 0.14	1.02 ± 0.13	0.166
Urea (mg/dL)	45.95 ± 15.24	43.29 ± 14.65	0.219

**Table 13:** Relation between diastolic dysfunction and echocardiographic findings.

		Diastolic Dysfunction		*p* Value
		Yes (*n* = 82)	No (*n* = 118)	
EF (%)		58.37 ± 4.83	59.91 ± 5.09	0.031
LAVI (mL/m^2^)		32.71 ± 4.48	31.25 ± 2.99	0.007
Septal e′ (m/s)		0.78 ± 0.18	0.87 ± 0.16	<0.001
Lateral e′ (m/s)		0.11 ± 0.04	0.13 ± 0.06	0.010
E/e′		9.37 ± 2.14	8.42 ± 1.5	<0.001
E/A		1.48 ± 0.49	1.33 ± 0.4	0.016
TR velocity (m/s)		1.92 ± 0.64	1.8 ± 0.58	0.166
DT (ms)		182.72 ± 40.74	204.94 ± 34.23	<0.001
IVRT (ms)		100.66 ± 18.69	112.95 ± 1 8.89	<0.001
Diastolic function grade	Normal	0 (0%)	39 (33.1%)	<0.001
	Grade I	23 (28%)	59 (50%)	
	Grade II	41 (50%)	17 (14.4%)	
	Grade III	18 (22%)	3 (2.5%)	
LVH		17 (20.7%)	20 (16.9%)	0.498

**Table 14:** Relation between CACS and baseline characteristics.

		CACS			*p* Value
		0 (*n* = 39)	1–100 (*n* = 84)	>100 (*n* = 77)	
Age (years)		51.6 ± 8.22	54.24 ± 9.18	55.71 ± 8.93	0.063
Sex	Male	19 (48.72%)	41 (48.81%)	44 (57.14%)	0.515
	Female	20 (51.28%)	43 (51.19%)	33 (42.86%)	
Weight (kg)		76.23 ± 11.53	73.61 ± 12.75	75.45 ± 12.6	0.475
Height (m)		1.68 ± 0.04	1.67 ± 0.05	1.68 ± 0.05	0.409
BMI (kg/m^2^)		27.03 ± 4.4	26.44 ± 4.71	26.9 ± 4.96	0.750

**Table 15:** Relation between CACS and risk factors.

	CACS			*p* Value
	0 (*n* = 39)	1–100 (*n* = 84)	>100 (*n* = 77)	
Smoking	10 (25.64%)	16 (19.05%)	26 (33.77%)	0.104
Family history	9 (23.08%)	11 (13.1%)	23 (29.87%)	0.034
Hypertension	13 (33.33%)	34 (40.48%)	33 (42.86%)	0.608
DM	10 (25.64%)	23 (27.38%)	24 (31.17%)	0.787
Dyslipidemia	11 (28.21%)	29 (34.52%)	26 (33.77%)	0.773

**Table 16:** Relation between CACS and laboratory investigations.

	CACS			*p* Value
	0 (*n* = 39)	1–100 (*n* = 84)	>100 (*n* = 77)	
Hb (g/dL)	12.47 ± 1.3	12.17 ± 1.08	12.44 ± 1.09	0.209
WBCs (×10^9^/L)	8.32 ± 2.09	8.26 ± 1.95	7.91 ± 1.98	0.435
Platelets (×10^9^/L)	288.28 ± 42.91	281.21 ± 43.36	277.59 ± 44.28	0.457
HbA1c (%)	5.15 ± 1.55	5.22 ± 1.48	5.28 ± 1.53	0.903
ALT (U/L)	32.08 ± 7.82	33.04 ± 8.46	30.75 ± 8.44	0.224
AST (U/L)	32.63 ± 6.66	32.73 ± 8	33.62 ± 7.28	0.696
Cholesterol (mg/dL)	206.88 ± 38.82	26.3 ± 58.18	243.05 ± 62.9	0.005
	*p*1 = 0.179, *p*2 = 0.004, *p*3 = 0.153			
TG (mg/dL)	193.03 ± 28.8	193.4 ± 34.24	202.37 ± 39.2	0.211
LDL (mg/dL)	103.23 ± 23.3	110.24 ± 23.93	116.18 ± 24.69	0.022
	*p*1 = 0.286, *p*2 = 0.018, *p*3 = 0.266			
HDL (mg/dL)	42.4 ± 4.97	40.6 ± 5.86	38.53 ± 5.25	0.001
	*p*1 = 0.200, *p*2 = 0.001, *p*3 = 0.0.046			
Creatinine (mg/dL)	1.01 ± 0.13	1.03 ± 0.14	1.04 ± 0.14	0.680
Urea (mg/dL)	43.38 ± 14.46	43.73 ± 13.95	45.63 ± 16.24	0.647

**Table 17:** Relation between CACS and echocardiographic findings.

		CACS			*p* Value
		0 (*n* = 39)	1–100 (*n* = 84)	>100 (*n* = 77)	
EF (%)		59.78 ± 5.23	59.12 ± 5.32	59.18 ± 4.62	0.780
LAVI (mL/m^2^)		30.8 ± 2.59	32.12 ± 3.78	33.17 ± 4.46	0.007
		*p*1 = 0.180, *p*2 = 0.005, *p*3 = 0.200			
Septal e′ (m/s)		0.87 ± 0.17	0.85 ± 0.17	0.79 ± 0.18	0.017
		*p*1 = 0.871, *p*2 = 0.039, *p*3 = 0.043			
Lateral e′ (m/s)		0.13 ± 0.06	0.12 ± 0.06	0.1 ± 0.05	0.014
		*p*1 = 0.537, *p*2 = 0.018, *p*3 = 0.091			
E/e′		8.33 ± 1.22	8.85 ± 1.79	9.45 ± 2.25	0.009
		*p*1 = 0.328, *p*2 = 0.008, *p*3 = 0.118			
E/A		1.26 ± 0.34	1.39 ± 0.45	1.46 ± 0.47	0.075
TR velocity (m/s)		1.64 ± 0.57	1.88 ± 0.58	2.07 ± 0.68	0.002
		*p*1 = 0.106, *p*2 = 0.001, *p*3 = 0.137			
DT (ms)		207.83 ± 33.07	197.15 ± 38.65	185.55 ± 40.2	0.010
		*p*1 = 0.316, *p*2 = 0.009, *p*3 = 0.137			
IVRT (ms)		118.2 ± 18.2	108.5 ± 19.5	101.9 ± 18.5	<0.001
		*p*1 = 0.022, *p*2 < 0.001, *p*3 = 0.073			
Diastolic function grade	Normal	38 (97.44%)	1 (1.19%)	0 (0%)	<0.001
	Grade I	1 (2.56%)	81 (96.43%)	0 (0%)	
	Grade II	0 (0%)	1 (1.19%)	57 (74.03%)	
	Grade III	0 (0%)	1 (1.19%)	20 (25.97%)	
LVH		7 (17.5%)	17 (20.2%)	13 (17.1%)	0.864

**Table 18:** Relation between CACS and segment involvement score (SIS).

		CACS			*p* Value
		0 (*n* = 39)	1–100 (*n* = 84)	>100 (*n* = 77)	
SIS		0.28 ± 1.04	1.25 ± 0.77	4.25 ± 0.9	<0.001
		*p*1 < 0.001, *p*2 < 0.001, *p*3 < 0.001			
SIS	0	39 (100%)	15 (17.86%)	0 (0%)	<0.001
	1–2	0 (0%)	64 (76.19%)	4 (10.26%)	
	>2	0 (0%)	5 (5.95%)	73 (94.81%)	

**Table 19:** Univariate regression analysis for incidence of diastolic dysfunction.

Variable	Unadjusted OR	95% Confidence Interval for OR		*p* Value
		Lower Bound	Upper Bound	
Age (years)	1.066	1.001	1.131	0.032
Male gender	3.086	1.239	7.681	0.016
DM	2.889	1.049	7.211	0.023
HTN	2.621	1.041	6.481	0.039
EF (%)	0.939	0.812	0.915	0.033
E/e′	1.187	1.044	1.357	0.008
LAVI (mL/m^2^)	1.091	1.015	1.172	0.018
TR velocity (m/s)	1.554	1.039	2.512	0.029
SIS	1.215	1.050	1.279	0.021
CACS	1.611	0.899	1.879	0.001
Obstructive CAD	0.921	0.865	1.863	0.001

**Table 20:** Multivariate regression analysis for incidence of diastolic dysfunction.

Variable	Unadjusted OR	95% Confidence Interval for OR		*p* Value
		Lower Bound	Upper Bound	
Age (years)	1.095	1.01	1.186	0.027
Male gender	6.34	1.505	26.699	0.012
DM	10.401	2.027	53.356	0.005
HTN	5.062	1.118	22.919	0.035
EF (%)	0.956	0.88	1.038	0.286
E/e′	1.395	1.078	1.805	0.011
LAVI (mL/m^2^)	1.132	1.003	1.277	0.044
TR velocity (m/s)	1.067	0.426	2.675	0.895
SIS	1.096	0.942	1.226	0.041
CACS	1.280	0.740	1.575	0.012
Obstructive CAD	1.235	1.127	2.141	0.013

## Data Availability

The data presented in this study are available on request from the corresponding author. The data are not publicly available due to privacy or ethical restrictions.

## Abbreviations

ALT: Alanine Aminotransferase; ASE: American Society of Echocardiography; AST: Aspartate Aminotransferase; BMI: Body Mass Index; CABG: Coronary Artery Bypass Grafting; CAD: Coronary Artery Disease; CAD-RADS: Coronary Artery Disease Reporting and Data System; CBC: Complete Blood Count; CACS: Coronary Artery Calcium Score; CCTA: Coronary Computed Tomographic Angiography; CIMT: Carotid Intima–Media Thickness; CTCA: Computed Tomography Coronary Angiography; DM: Diabetes Mellitus; DT: Deceleration Time; E: Mitral Inflow Early Filling Velocity; E/A: Mitral Inflow Early to Late Filling Velocity Ratio; EACVI: European Association of Cardiovascular Imaging; EF: Ejection Fraction; FFR-CT: Fractional Flow Reserve by Computed Tomography; Hb: Hemoglobin; HbA1c: Glycated Hemoglobin; HDL: High-Density Lipoprotein; HFpEF: Heart Failure with Preserved Ejection Fraction; IQR: Interquartile Range; IVRT: Isovolumic Relaxation Time; LA: Left Atrium; LAVI: Left Atrial Volume Index; LDL: Low-Density Lipoprotein; LV: Left Ventricle; LVEF: Left Ventricular Ejection Fraction; LVH: Left Ventricular Hypertrophy; PCI: Percutaneous Coronary Intervention; SD: Standard Deviation; SIS: Segment Involvement Score; SPSS: Statistical Package for the Social Sciences; TG: Triglycerides; TR: Tricuspid Regurgitation; TTE: Transthoracic Echocardiography; WBCs: White Blood Cells.
